# Supplementary material for: Clostridioides difficile Infection Treatment and Outcome Disparities in a National Sample of United States Hospitals
Source: Antibiotics (Basel). 2022 Sep 6;11(9):1203. doi: 10.3390/antibiotics11091203 (PMC9495221; doi:10.3390/antibiotics11091203)
Supplement: Supplementary file 1 [file antibiotics-11-01203-s001.zip › antibiotics-1866601-supplementary.pdf]

Table S1. Selected CDI patient characteristics, treatment patterns, and outcomes by study year for index encounters

**Study year**	**2018 (n=14,563)**	**2019 (n=12,456)**	**2020 (n=9978)**	**2021 (n=1767)**
**Patient characteristics**				
Age, median (IQR)	68 (56-79)	68 (55-78)	68 (56-78)	68 (58-78)
Female sex, %	58.3	56.6	58.0	56.9
Race, %				
Black	12.1	12.4	12.5	13.3
Other	6.3	7.1	6.5	6.6
White	81.6	80.5	81.1	80.1
Hispanic ethnicity, %	3.8	5.4	8.0	6.3
**CDI treatments**				
CDI therapies, %				
Metronidazole	50.3	42.1	38.7	37.2
Vancomycin	70.3	75.7	80.3	82.6
Fidaxomicin	3.9	4.9	5.0	3.3
Bezlotoxumab	0.1	<0.1	<0.1	0.0
FMT	0.2	0.1	0.1	0.0
Costs (USD), median				
Hospital costs	10863	10534	12533	14216
Patient charges	42480	41933	48012	51504
**CDI outcomes**				
Severe CDI, %	41.0	41.2	43.2	44.1
CDI recurrence, %[a]	10.7	9.3	7.2	N/A
In-hospital mortality, %[b]	6.3	5.5	6.6	8.8
Hospital LOS, median (IQR)[b]	7 (4-13)	7 (4-13)	7 (4-14)	8 (5-16)

CDI=*Clostridioides difficile* infection; IQR=interquartile range

[a] Data represent patients who survived the index visit in 2018-2020 only

[b] Data represent hospitalized patients only
